# Phosphorylation of the Zebrafish M6Ab at Serine 263 Contributes to Filopodium Formation in PC12 Cells and Neurite Outgrowth in Zebrafish Embryos

**DOI:** 10.1371/journal.pone.0026461

**Published:** 2011-10-20

**Authors:** Kai-Yun Huang, Gen-Der Chen, Chia-Hsiung Cheng, Kuan-Ya Liao, Chin-Chun Hung, Geen-Dong Chang, Pung-Pung Hwang, Shu-Yu Lin, Ming-Chieh Tsai, Kay-Hooi Khoo, Ming-Ting Lee, Chang-Jen Huang

**Affiliations:** 1 Institute of Biochemical Sciences, National Taiwan University, Taipei, Taiwan; 2 Institute of Biological Chemistry, Academia Sinica, Taipei, Taiwan; 3 Institute of Cellular and Organismic Biology, Academia Sinica, Taipei, Taiwan; 4 Academia Sinica Common Mass Spectrometry Facilities at Institute of Biological Chemistry, Academia Sinica, Taipei, Taiwan; 5 National Research Program for Genomic Medicine (NRPGM) Core Facilities for Proteomics and Glycomcis at Institute of Biological Chemistry, Academia Sinica, Taipei, Taiwan; MRC, University College of London, United Kingdom

## Abstract

**Background:**

Mammalian M6A, a member of the proteolipid protein (PLP/DM20) family expressed in neurons, was first isolated by expression cloning with a monoclonal antibody. Overexpression of M6A was shown to induce filopodium formation in neuronal cells; however, the underlying mechanism of is largely unknown. Possibly due to gene duplication, there are two M6A paralogs, M6Aa and M6Ab, in the zebrafish genome. In the present study, we used the zebrafish as a model system to investigate the role of zebrafish M6Ab in filopodium formation in PC12 cells and neurite outgrowth in zebrafish embryos.

**Methodology/Principal Findings:**

We demonstrated that zebrafish M6Ab promoted extensive filopodium formation in NGF-treated PC12 cells, which is similar to the function of mammalian M6A. Phosphorylation at serine 263 of zebrafish M6Ab contributed to this induction. Transfection of the S263A mutant protein greatly reduced filopodium formation in PC12 cells. In zebrafish embryos, only S263D could induce neurite outgrowth.

**Conclusions/Significance:**

Our results reveal that the phosphorylation status of zebrafish M6Ab at serine 263 is critical for its role in regulating filopodium formation and neurite outgrowth.

## Introduction

The proteolipid protein (PLP), an integral membrane protein with four transmembrane domains, is abundant in the central nervous system [1]. DM20, an alternative splicing form of PLP, lacks a unique 35-amino acid segment [2]. Mouse M6A and M6B were first identified by expression cloning using an M6-20 monoclonal antibody. M6A is 43% and 56% identical to DM20 and M6B at the amino acid level [3]. Due to genome duplication, three pairs of PLP family members were identified in zebrafish, termed DMá1 and DMá2, DMâ1 and DMâ2, and DMã1 and DMã2 [4]. A gene expression pattern analysis revealed that DMâ and DMã are neuronal glycoproteins, whereas DMá/PLP/DM20 are myelin proteins. DMá1 is respectively 59% and 60% identical to human DM20 and DMá2 at the amino acid level, while DMá2 is only 49% identical to human DM20. In contrast, both DMâ1 and DMâ2 show a higher identity of 85% with human and mouse M6A and are also respectively called M6Aa and M6Ab. Similarly, DMã2 is 81% identical to human M6B and 83% to DMã1 at the amino acid level. In mammals, M6A is present in neurons, while M6B is found in both neurons and glia [5].

M6A was first isolated by expression cloning with a monoclonal antibody [3], and treatment of this antibody was found to interfere with neurite extension of cultured cerebellar neurons [6]. These data suggest that M6A may play an important role in controlling nerve extension. Indeed, overexpression of M6A in cultured primary hippocampal neurons promotes neurite outgrowth and the formation of filopodial protrusions [7]. Although the mechanism of action of M6A is still largely unknown, M6A was shown to be involved in a number of biological processes. For example, Ca^2+^ influx is increased by the overexpression of M6A in nerve growth factor (NGF)-treated rat pheochromocytoma PC12 cells [8]. M6A was also found to bind to the μ-opioid receptor and facilitate receptor endocytosis and recycling [9]. Moreover, expression of the M6A transcript decreased under pathological conditions such as chronic stress in animals and depression in humans [7].

Structurally, M6A is a glycoprotein with four transmembrane domains, which form one intracellular (IC) and two extracellular (EC) loops. Both the N- and C-terminal regions are located in the cytoplasm [3], [4], [10]. Several studies identified the region or the phosphorylation site within M6A that is critical for neurite/filopodium outgrowth. Mutation analysis of two cysteine residues (C44 and C46) in EC1 and four cysteine residues (C162, C174, C192, and C202) in EC2 provided important data that neurons expressing C174A and/or C192A mutants display decreased filopodium numbers [10]. This suggests that cysteine residues in the EC2 domain of M6A play important roles in filopodium outgrowth. On the other hand, there are one putative phosphorylation site for casein-kinase 2 (CK2), i.e., S256, and two for protein kinase C (PKC), i.e., S267 and T268, in the C-terminal region of rat M6A. Two of these sites (S256 and S267) were identified by phosphoproteomic studies of brain tissues [11], [12]. Moreover, expression of neither S256A nor the S267A/T268A mutant protein of M6A in primary hippocampal neurons affected their ability to promote filopodium formation, but did affect protrusion motility [13].

In this study, we demonstrate that zebrafish M6Ab can induce high-density filopodium formation in NGF-treated PC12 cells, which is similar to the function of mammalian M6A [7]. This is not surprising because zebrafish M6Ab is 85% identical to rat M6A [4]. However, phosphorylation at serine 263 of zebrafish M6Ab, which corresponds to serine 256 of rat M6A, contributes to this induction. Transfection of the S263A mutant protein greatly reduced filopodium formation in PC12 cells. Interestingly, only S263D, but not the wild-type (WT) M6Ab, could induce neurite outgrowth in zebrafish embryos, suggesting that WT M6Ab requires further activation by other signal pathway.

## Results

### Zebrafish M6Ab is an N-linked glycoprotein

**Figure 1:**
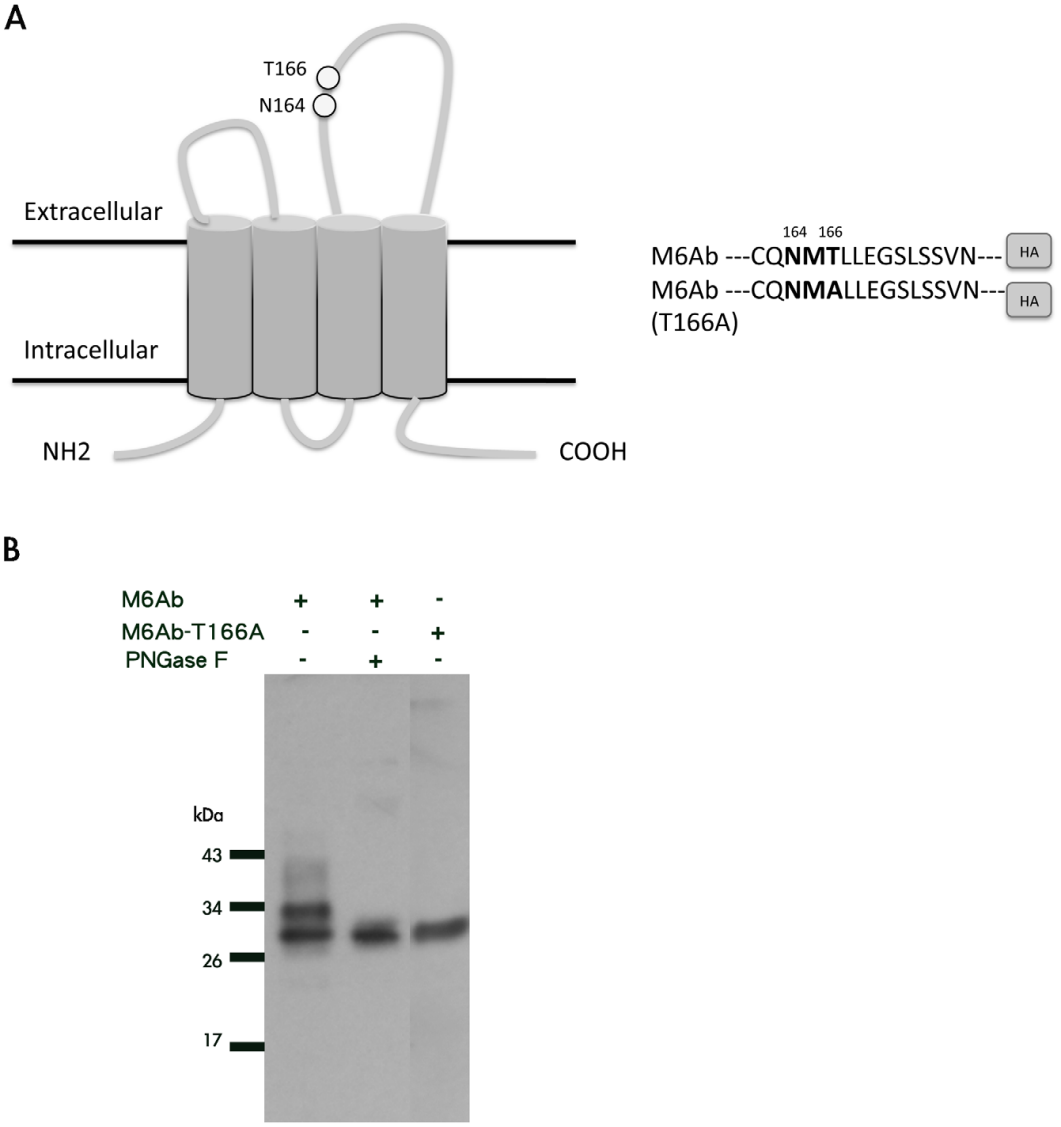
Zebrafish M6Ab is an N-linked glycoprotein. (A) M6Ab structural features. Based on the predicted computational model, zM6Ab contains four transmembrane domains [10]. The proposed N-glycosylation sites, NXT (164∼166), are localized to the second extracellular domain. (B) COS-1 cells were transfected with the pcDNA3-M6Ab-HA and pcDNA3-M6Ab (T166A)-HA plasmids. Two days after transfection, cell lysates were treated with PNGase F for 18 h at 37°C and then subjected to an SDS-PAGE and Western blot analysis using an anti-HA antibody.

M6A was identified as a glycoprotein in the mouse, rat, and human and contains two potential N-glycosylation sites, ^164^NTT and ^208^NMT, in the EC2 region of rat M6A [10]. In zebrafish, only one potential N-glycosylation site, ^164^NMT, was found in the EC2 region of M6Ab (Fig. 1A). Zebrafish M6Ab protein expressed in COS-1 cells appears in SDS-PAGE as two major bands with apparent molecular masses of approximately 28 and 32 kDa (Fig. 1B). After enzymatic digestion with peptide N-glycosidase (PNGase)-F, which removes both high-mannose, hybrid- and complex-type N-linked glycans, we observed that the mature form of zebrafish M6Ab displayed faster electrophoretic mobility. This result indicated that zebrafish M6Ab contains high-mannose and/or complex-type N-glycans. In order to investigate the role of N-linked glycans in the function of zebrafish M6Ab, the potential N-glycosylation site, ^164^NMT, was mutated to ^164^NMA, which was also designed as T166A. COS-1 cells were transfected with the pcDNA3-HA vector coding for M6Ab-wt or the T166A mutant. HA-tagged recombinant protein expression was analyzed by immunoblotting, using a mouse anti-HA tag antibody. The T166A mutant was detected as a protein with only a smaller molecular size, which was the same as that of WT M6Ab after treated with PNGase-F. Taken together, these results indicate that M6Ab is an N-glycosylated glycoprotein.

### Overexpression of zebrafish M6Ab induces neurite outgrowth in PC12 cells and filopodium formation in both COS-1 and PC12 cells

**Figure 2:**
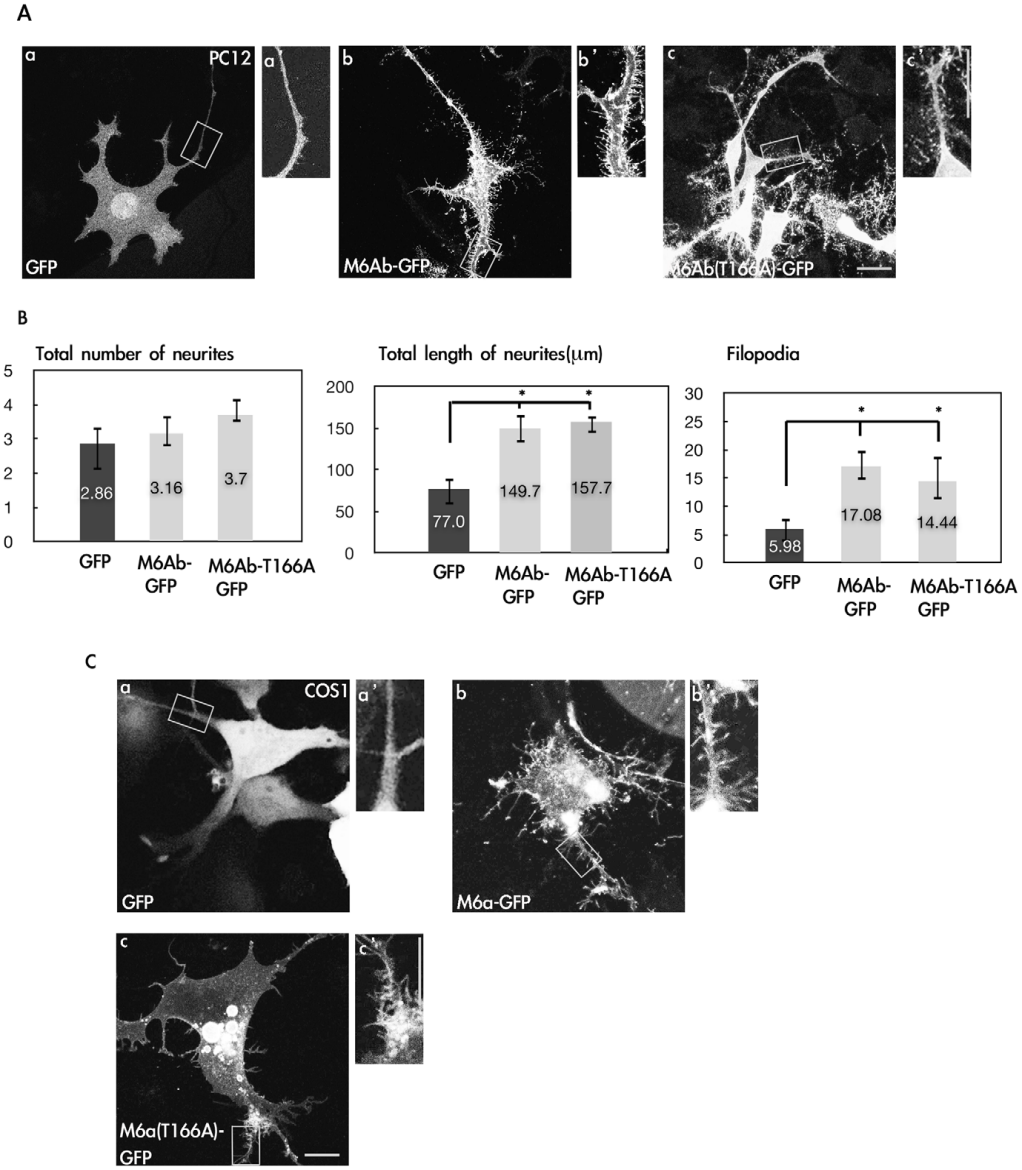
Overexpression of zM6Ab can induce neurite outgrowth and filopodia in PC12 and COS-1 cells. (A) PC12 cells were transfected with the control pcDNA3-GFP or pcDNA3-M6Ab-GFP or pcDNA3-M6Ab-T166A-GFP plasmids. Twenty-four hours after transfection, cells were treated with nerve growth factor (NGF) (100 ng/ml) for 2 days. Cells were then fixed, and images were taken with a Zeiss LSM510 laser scanning confocal microscope. The insets are the 2× magnified images of the boxed areas. (B) Quantification of the total number of neurites, total length of neurites, and filopodium-like processes in a 20-µm neurite length. * indicates a significant difference compared with the respective control of GFP (P<0.05). (C) COS-1 cells were transfected with the control pcDNA3-GFP or pcDNA-M6Ab-GFP or pcDNA3-M6Ab-T166A-GFP plasmids. Cells were fixed, and images were taken with a Zeiss LSM510 laser scanning confocal microscope. Scale bars, 10 µm.

To investigate the possible function of zebrafish M6Ab and the cellular consequences of M6Ab overexpression, we used PC12 cells, a well-defined cell model system which is widely used in studies of neuritogenesis. We first expressed M6Ab fused to the green fluorescence protein (GFP) or GFP alone in PC12 cells to assess whether it modulates filopodium formation. Overexpression of zebrafish M6Ab-GFP promoted filopodium formation and neurite outgrowth in NGF-treated PC12 cells compared to GFP alone (Fig. 2A, B), and this result was similar to the expression of mouse and rat M6A in hippocampal neurons [7]. In addition, zebrafish M6Ab-GFP was found to promote filopodium formation in non-neuronal cell lines such as COS-1 (Fig. 2C). However, the glycosylation mutant, T166A, as mentioned in Fig. 1, showed similar effects on the filopodium formation in either PC12 or COS-1 cells (Fig. 2A, panels c and c′; 2B and 2C, panels c and c′). These results suggest that zebrafish M6Ab, glycosylated or not, can promote filopodium formation in both neuronal-like and non-neuronal cell lines.

### Serine residue S263 is critical for M6Ab-induced filopodium formation in PC12 cells

It was recently reported by Dr. A. C. Frasch's group that overexpression of M6A induces neurite formation and increases filopodium density in hippocampal neurons and neuroblastoma N2a cells [7], [10]. Although the identity of upstream kinase of M6A remains unknown, we observed that M6A-induced neurite formation was blocked when PC12 cells were treated with a PKC inhibitor [8]. This suggests that PKC may act as a potential upstream protein kinase for M6A. Similar to rat M6A, zebrafish M6Ab has two putative phosphorylation sites, S274 and S277, by PKC and one site, S263, for casein kinase 2 (CK2). Those three serine residues are located in the C-terminal region of zebrafish M6Ab [4]. To further investigate whether M6Ab C-terminal phosphorylation contributes to the regulation of neurite outgrowth and filopodium formation, several mutant proteins aimed at those three serine phosphorylation sites were generated by site-directed mutagenesis. An alanine or aspartic acid residue was introduced to replace the original serine residue to mimic the unphosphorylated or constitutively active form. We also generated the triple mutants S263A/274A/S277A (A3) and S263D/274D/S277D (D3) to further elucidate the critical roles of these three serine residues.

**Figure 3:**
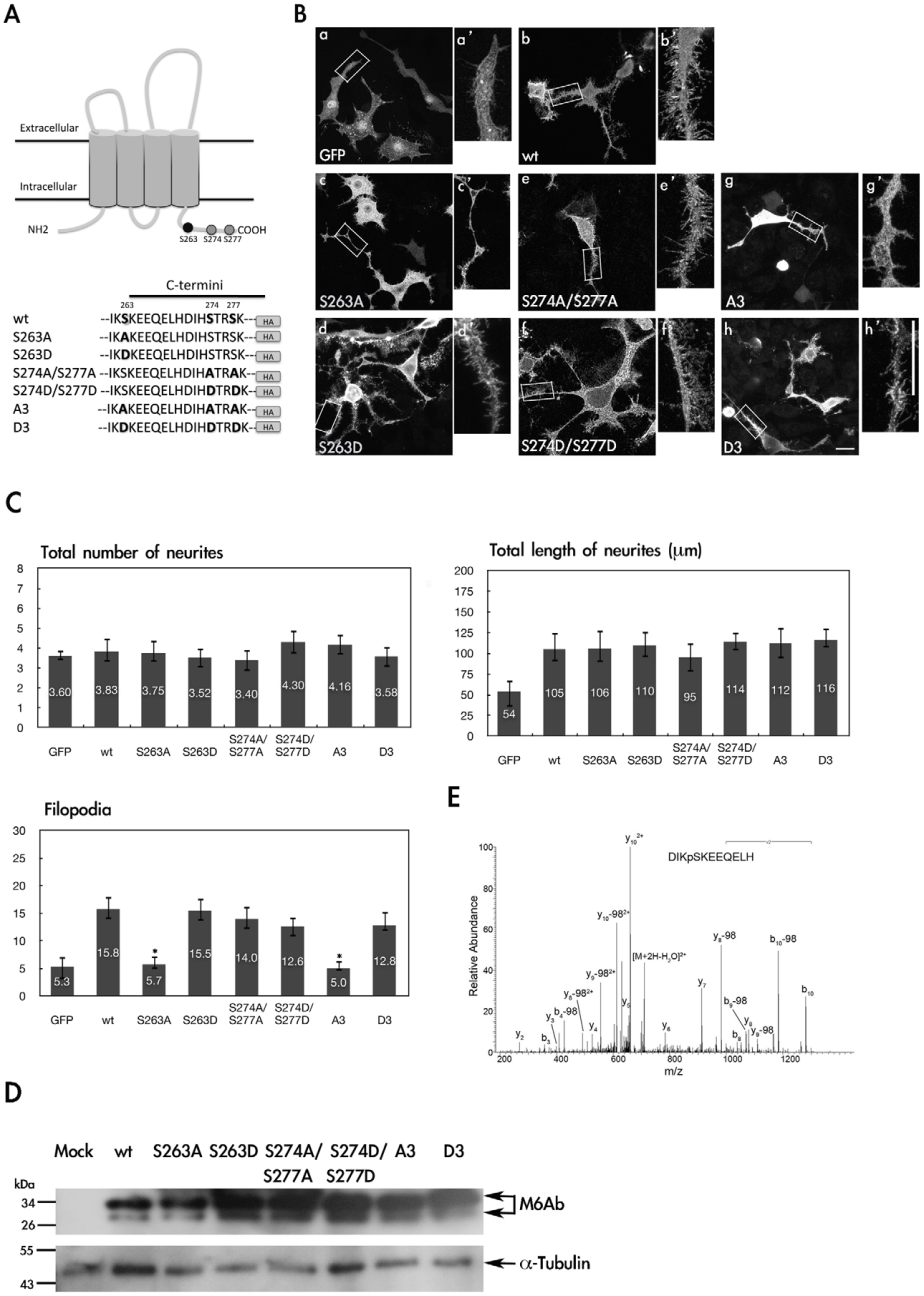
Phosphorylation of S263 is critical for regulation of filopodium formation in PC12 cells. (A) Partial amino acid sequences of the wide-type and mutant proteins of zebrafish M6Ab. (B) PC12 cells were transfected with pcDNA3-GFP-HA(a), pcDNA3-M6Ab-HA(b), pcDNA3-M6Ab(S263A)-HA(c), pcDNA3-M6Ab (S263D)-HA(d), pcDNA3-M6Ab(S274A/S277A)-HA(e), pcDNA3-M6Ab(S274D/S277D)-HA(f), pcDNA3-M6Ab(A3)-HA(g), or pcDNA3-M6Ab(D3)-HA(h) plasmids. Twenty-four hours after transfection, cells were treated with nerve growth factor (NGF) (100 ng/ml) for 2 days. Then transfected cells were fixed, and M6Ab-HA was detected using anti-HA antibodies for immunostaining. PC12 neurites are shown at a higher magnification (a′–h′). (C) Quantification of the total number of neurites, total length of neurites, and filopodium-like processes in a 20- · m neurite length. Results are expressed as the mean ± SD of at least 40∼50 neurites. At least three independent experiments were analyzed. *Significant difference compared with the respective control of WT-M6Ab overexpression (P<0.05). (D) Cell lysates from different transfected cells as indicated were extracted and immunoblotted with an anti-HA antibody or anti-Tubulin antibody. (E) MS/MS spectrum on [M+2H]^2+^ (m/z 718.32) ion for the peptides DIKpSKEEQELH from WT M6Ab protein. The product ion y_8_ which carries a phosphate indicated that Serine 263 was phosphorylated. Residues bearing phosphate moieties are indicated with p. “b” and “y” ion series represent fragment ions containing the N- and C-termini of the peptide, respectively. Scale bars, 10 µm.

PC12 cells were first transiently transfected with different expression plasmids encoding mutant proteins, such as S263A or S263D, S274A/S277A or S274D/S277D, and A3 or D3. Transfected cells were then treated with NGF to induce neuronal differentiation in order to detect subcellular localizations using immunostaining and immunofluorescence microscopy (Fig. 3A). Expression levels of each mutant protein and WT M6Ab were checked by immunoblotting with an anti-HA monoclonal antibody. The membrane was also stripped and reprobed with antibodies against tubulin (Fig. 3D). The expression level of either the S263A mutant or the triple A3 mutant was less than that of the other four groups, but only the S263A mutant and the triple A3 mutant greatly reduced filopodium formation.

Our data revealed that WT M6Ab was localized to membrane protrusions (filopodia) (Fig. 3B, panels b and b′), and the extent of filopodium formation and neurite growth was very obvious (Fig. 3C). Overexpression of the S263A mutant (Fig. 3B, panels c and c′) or the triple mutant, S263A/274A/S277A (Fig. 3B, panels h and h′), greatly reduced filopodium formation, while neurite outgrowth or neurite numbers in NGF-treated PC12 remained unchanged compared to WT M6Ab (Fig. 3C). The filopodium numbers of the S263A mutant or the triple mutant were reduced to be the same as those in GFP-transfected PC12 cells. On the other hand, the S274A/S277A double mutant caused little change in the relative abundance of neurite outgrowth, neurite numbers, or filopodium formation (Fig. 3B, panels e and e′). These data clearly suggest that S263 plays an important role in regulating filopodium formation in NGF-induced PC12 cells. Due to the high-density filopodium formation by WT M6Ab, it was difficult to analyze whether or not filopodium formation had increased with those constitutively active forms, such as S263D or S263D/274D/S277D (D3) (data not shown).

We also used nano liquid chromatography–mass spectrometry analysis to confirm the phosphorylation at Serine 263 in WT M6Ab. As shown in Figure 3E, a phosphorylated peptide from WT M6Ab protein was identified as DIKpSKEEQELH due to the observed ions y_8_ which carries a phosphate. This indicated the phosphorylation site was at Serine 263.

Taken together, these results suggest that the S263 residue of M6Ab greatly contributes to the regulation of filopodium morphogenesis.

### CaMKII and MEK1/2 may be involved in zebrafish M6Ab-induced early neurite outgrowth in NGF-treated PC12 cells

**Figure 4:**
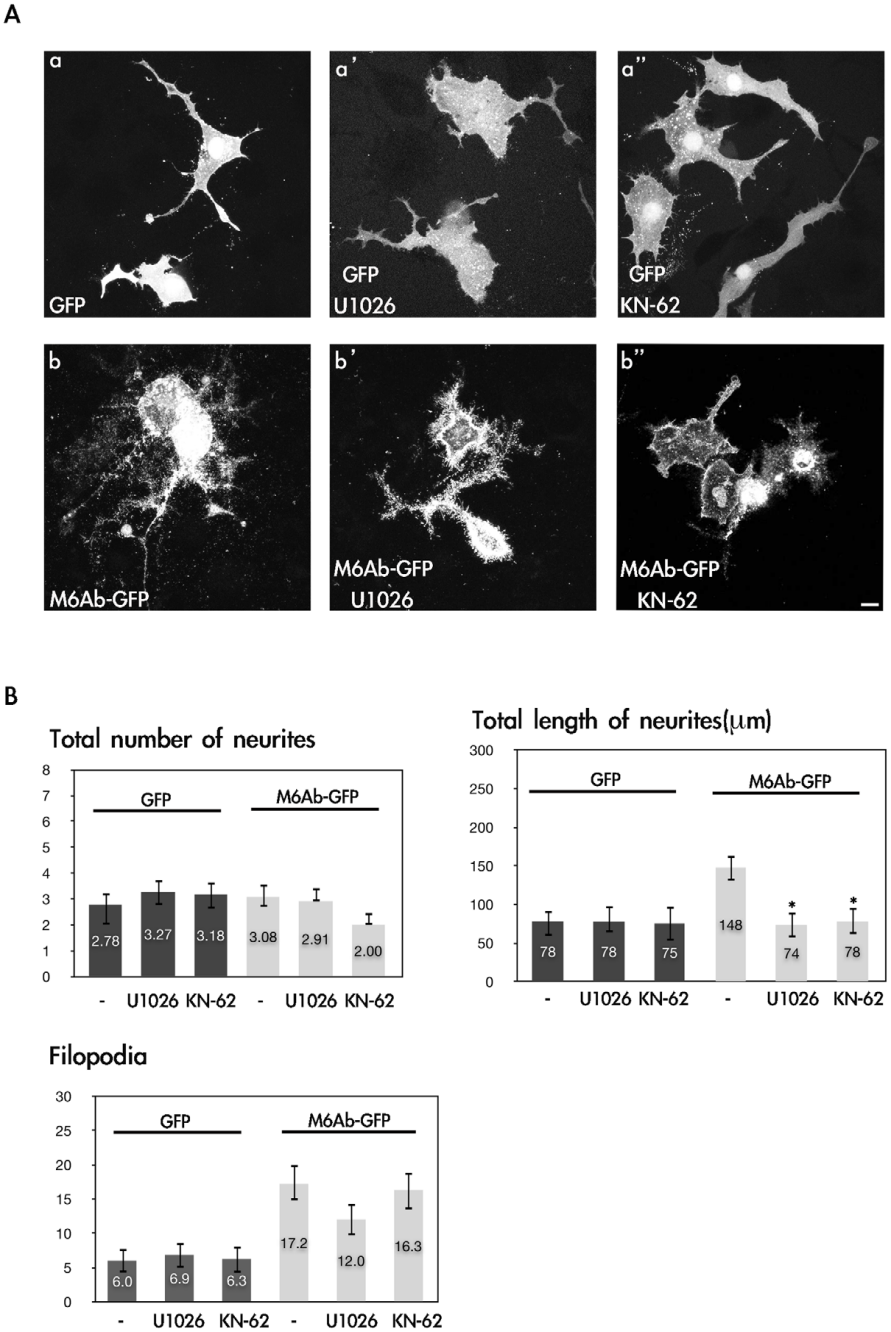
Inhibition of CaMKII and MEK1/2 reduces M6Ab early neurite outgrowth in nerve growth factor (NGF)-differentiated PC12 cells. (A) PC12 cells were transfected with the pcDNA3-M6Ab-GFP or pcDNA3-GFP plasmids. After transfection, PC12 cells were differentiated with 100 ng/ml NGF for 72 h in the presence of 10 µM KN-62 or 10 µM U1026 (Con; 0.1% DMSO). Cells were fixed, and images were taken with a Zeiss LSM510 laser scanning confocal microscope. (B) Quantification of the total number of neurites, total length of neurites, and filopodium-like processes in a 20-µm neurite length. Results are expressed as the mean ± SD of at least 40∼50 neurites. At least three independent experiments were analyzed. *Significant difference compared with the respective control of WT M6Ab overexpression (P<0.05). Scale bars, 10 µm.

As mentioned above, overexpression of M6Ab in PC12 cells resulted in the promotion of neurite outgrowth and filopodium formation (Fig. 2A). Several studies demonstrated that dendritic filopodia are capable of participating in synapse formation [14], [15], and filopodium formation was suggested to play a permissive role in synaptogenesis [16]. However, the underlying mechanism of how M6Ab regulates neurite outgrowth and filopodium formation remains unclear. As shown in Fig 3, phosphorylation of serine 263 is crucial for M6Ab's ability to promote filopodium formation in PC12 cells. To further confirm which kinase contributes to the phosphorylation of M6Ab at serine 263 and to analyze the signaling pathway involved in M6Ab-induced neurite outgrowth and filopodium formation, two kinase inhibitors were utilized. In general, PC12 cells were first transfected with pcDNA-GFP alone or pCDNA-M6Ab-GFP, then treated with a different inhibitor, such as U0126 (for MEK1/2 that prevents activation of MAPK by MEK) or KN-62 (a CaMKII inhibitor) in the presence of NGF. No significant difference was found between untreated GFP-expressing PC12 cells compared to GFP-expressing PC12 cells treated with U0126 or KN-62 (Fig. 4A, panels a and a′). Both U0126 and KN-62 significantly reduced the outgrowth of M6Ab-induced neurite outgrowth, but had no effect on M6Ab-induced filopodium formation in PC12 cells (Fig. 4B). In addition, we also tested PKC inhibitors, such as Gö6983 and Ro-31-8425, in the same way and found no effect on M6Ab-induced filopodium formation in PC12 cells (data not shown). These results indicated that CaMKII kinases, MAPK and PKC may not participate in M6Ab-induced filopodium formation in PC12 cells.

### M6Ab can induce neurite outgrowth in the presence of constitutively active CaMKIIβ1 in zebrafish embryos

**Figure 5:**
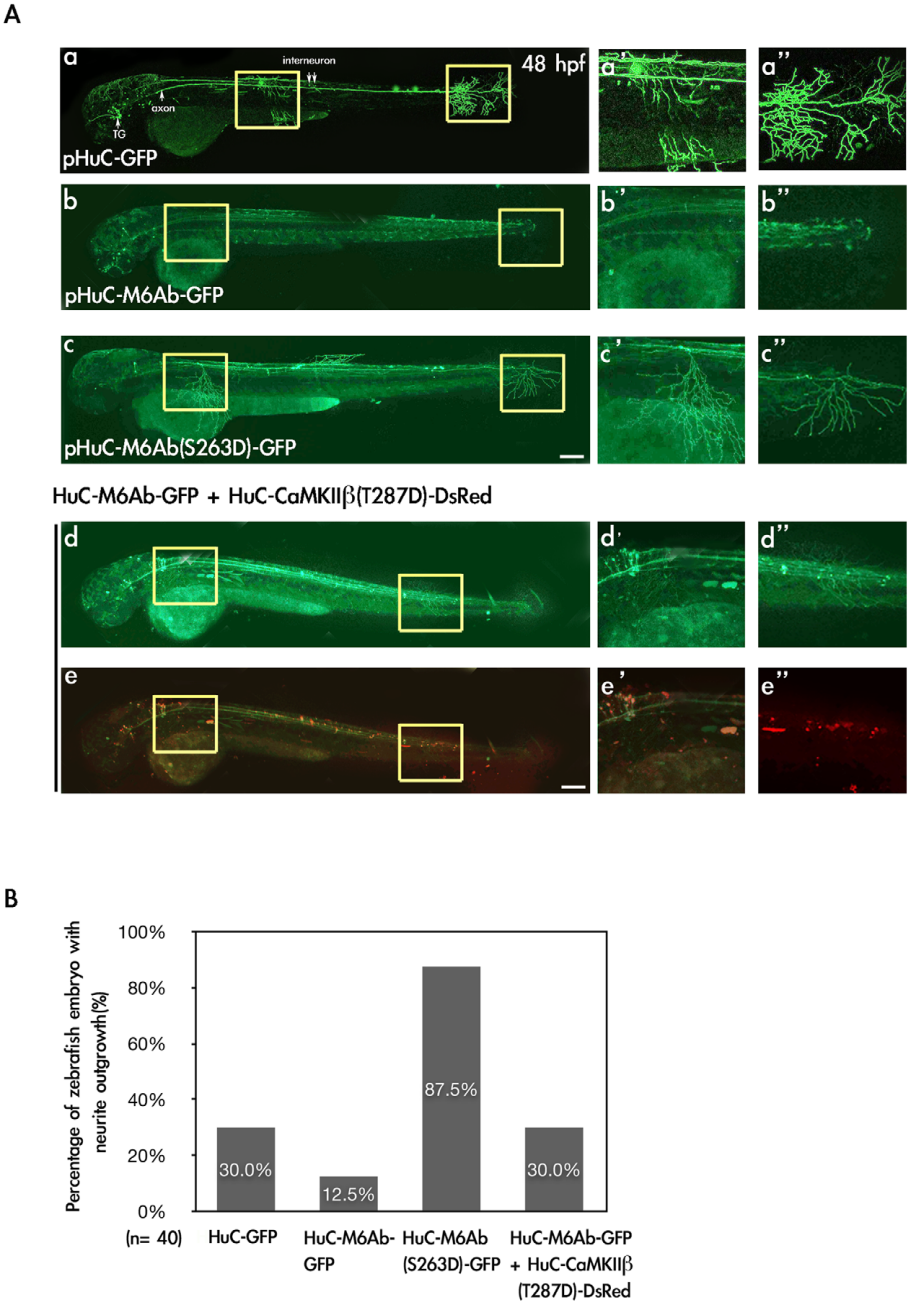
Expression of M6Ab-GFP and M6Ab(S263D)-GFP driven by a neuron-specific HuC promoter in zebrafish embryos. (A) To express the wild-type and S263D mutant proteins of zebrafish M6Ab under the control of a neuron-specific HuC promoter, plasmids pHuC-GFP, pHuC-M6Ab-GFP, pHuC-M6Ab(S263D)-GFP, and pHuC-M6Ab-GFP/pHuC-CaMKII · 1(T287D)-DsRed were individually injected into zebrafish embryos at the one-cell stage. Zebrafish embryos at 48 h post-fertilization (hpf) with GFP fluorescence were selected for the image analysis. Images were taken using a Zeiss LSM510 laser scanning confocal microscope. Merged images of red and green fluorescence are shown in (e, e′, and e″), while only green fluorescence images are shown in (a, a′, a″, b, b′, b″, c, c′, c″, d, d′ and d″). Higher magnification of two regions marked with yellow boxes in panel a-e is shown in panels a′–e′ and a″–e″, respectively. (B) Quantification of zebrafish numbers with significant neurite outgrowth at 48 hpf. Scale bars, 10 µm.

Like mammalian M6A, zebrafish M6Ab was also found to be capable of inducing high-density filopodium formation in NGF-treated PC12 cells (Fig. 2A). To further elucidate whether zM6Ab can trigger neurite outgrowth in zebrafish embryos, the GFP fusion protein of WT zM6Ab was expressed under the control of a neuron-specific HuC promoter in zebrafish embryos. The *HuC* gene is known as a useful early marker for neurons in zebrafish, and the upstream 3.4 kb-long promoter fragment was demonstrated to be sufficient to confer on its downstream target gene a neuron-specific expression pattern closely resembling that of the endogenous *HuC* gene. As shown in Fig. 5A, *HuC* promoter-driven GFP was expressed in trigeminal ganglia, axons, and interneurons of zebrafish embryos at 48 hpf (Fig. 5A, panel a) [17], [18], [19]. The expression pattern of zM6Ab-GFP was observed to be similar to that of GFP (Fig. 5A, panels b, b′ and b″), but the percentage of zebrafish embryo with neurite outgrowth was only 12.5% compared to 30% of the control zebrafish injected with pHuC-GFP (Fig. 5B). However, the S263D mutant protein induced significant neurite outgrowth with neurites covering the yolk ball or reaching the margin of the dorsal and ventral fins (Fig. 5A, panels c, c′, c″). The percentage of zebrafish embryo with neurite outgrowth reached 87.5% (Fig. 5B). These data suggest that zM6Ab needs to be activated by another signaling pathway to induce neurite outgrowth in zebrafish embryos during development.

Based on the phosphorylation site consensus sequences (KinasePhos 2.0 program; http://kinasephos2.mbc.nctu.edu.tw) [20], the Motif (^263^ SKEE) is a potential target sequence for casein kinase II (CKII) and calcium/calmodulin-dependent protein kinase II (CaMKII). Therefore, we further examined whether CaMKII activity could trigger neurite outgrowth by zM6Ab-GFP in zebrafish embryos. A constitutively active form of zebrafish CaMKII · β1 (T287D) (pHuC-CaMKII · β1 (T287D)-DsRed) was generated by site-directed mutagenesis and then driven by the same neuron-specific HuC promoter with a second reporter *DsRed* gene. Plasmid DNAs of both pHuC-CaMKII β1 (T287D)-DsRed and pHuC-M6Ab-GFP were co-injected into zebrafish embryos at the 1-cell stage. Although neurite outgrowth by zM6Ab-GFP was observed in zebrafish embryos at 48 hpf (Fig. 5A, panels d, d′, d″), but the percentage of zebrafish embryo with neurite outgrowth was only 30% (Fig. 5B). These data indicate that the wild-type M6Ab possibly inhibited the neurite outgrowth, and co-expression of CaMKIIβ1(T287D) could only restore the neurite outgrowth to the normal level, 30%. Again, the phosphorylation status of zM6Ab is critical for neurite outgrowth in vivo and which protein kinase is involved to regulate the phosphorylation of zM6Ab at serine 263 in zebrafish embryos needs further investigstion.

## Discussion

Due to genome duplication, there are two M6A homologs, DMâ1 and DMâ2, in the zebrafish genome [4]. Both DMâ1 and DMâ2 show an identity of 85% at the amino acid level to M6A of the human, mouse, and rat. Due to correlations with the function of mammalian M6A, we adopted the names, M6Aa and M6Ab, used in the databank, instead of DMâ1 and DMâ2. Human M6A is a protein of 267 amino acids which lacks the N-terminal 11 amino acid residues, found in zM6Aa, zM6Ab, mouse M6A, and rat M6A. There is a stretch of 7 amino acid residues, ^171^SLSSVNS, which is only present in zM6Ab. Without these residues, zM6Ab exhibits 87% identity to zM6Aa. In this study, we provide evidence indicating that zebrafish M6Ab may induce high-density filopodium formation in NGF-treated PC12 cells (Fig. 2), and its phosphorylation at serine 263 (^263^SKEE) possibly contributes to this induction, as expression of the S263A mutant protein greatly reduced filopodium formation in PC12 cells (Fig. 3). Interestingly, zM6Aa may also induce high-density filopodium formation in NGF-treated PC12 cells (data not shown), but its corresponding serine residue (^256^SKEE) is not essential for filopodium formation in PC12 cells. However, the S256A mutant protein of zM6Aa still has the ability to induce filopodium formation in PC12 cells (data not shown). These data contain similarities to a recent report that expression of the S256A mutant protein of rat M6A in primary hippocampal neurons does not affect its ability to promote filopodium formation, although it may affect protrusion motility [13]. Although we did not perform protrusion motility studies, zM6Aa is a possible ortholog of mammalian M6A. Further investigations are pending, in order to further explore the relationships of structure and function between zM6Aa and zM6Ab.

Gene duplication in the zebrafish genome is commonly observed and results from an early duplication specific to ray finned fish [21]. Based on molecular phylogenetic and gene synteny analyses, two duplicated genes were proposed to have emerged as a consequence of whole-genome duplication before the divergence of jawed vertebrates. The retention of functional gene duplicates in genomes, known as paralogs, is attributed to their role in maintaining a prompt response when loss-of-function mutations in one copy of an essential gene occur [21], [22]. Neofunctionalization may follow [23], [24]. As a result, paralogs may evolve to have distinct expression patterns and functions.

Mammalian M6A was shown to induce neurite outgrowth in cultured primary hippocampal neurons [7], but whether it can also promote neurite outgrowth in vivo is unclear. In the present study, we showed that only the S263D mutant protein, not the WT M6Ab, could induce neurite outgrowth in zebrafish embryos (Fig. 5A, panels b and c). On the other hand, the WT M6Ab could promote neurite outgrowth only in the presence of a constitutively active CaMIIβ1 (panels d and e). These data suggest that WT M6Ab can be activated through other signal pathways to induce neurite outgrowth in zebrafish embryos. One of these pathways is mediated by type II calcium-calmodulin activated protein kinase (CaMKII). This is consistent with an earlier notion that mammalian M6A can act as an NGF-gated Ca^2+^ channel in neuronal differentiation, and Ca^2+^ influx increases when M6A is overexpressed in NGF-treated rat pheochromocytoma PC12 cells [8]. However, it is difficult to envision that M6A functions as a calcium channel, M6A may regulate the expression or function of calcium channels instead. It may be worth investigating whether calcium influx is affected when the expression of either zM6Aa or zM6Ab is knocked down by morpholino oligonucleotide (MO) technology [25]. In *zM6Ab* MO-injected zebrafish embryos, we can further test whether the expression of *zM6Aa* mRNA can rescue its phenotype. Such experiments will clarify whether *zM6Aa* and *zM6Ab* are redundant genes or perhaps have different roles during zebrafish development.

An increase in the Ca^2+^ concentration in response to extracellular stimuli can activate various Ca^2+^/calmodulin (CaM)-dependent enzymes including Ca^2+^/CaM-dependent protein kinases (CaMKs) to regulate a variety of cellular processes [26], [27]. Among many serine/threonine CaM kinases, CaMK-II is known for its high concentration in the adult central nervous system [28] with vital roles in spatial memory [29], [30] and neuron function [31]. In mammals, CaMK-II is composed of four isoforms, α, β, γ, and δ. Each isoform has several alternative spliced forms [32], [33]. As a result of genome duplication, four pairs of the CaMK-II family member were identified in zebrafish [34]. Similarly, at least 20 splice variants were found to have been generated by alternative splicing during development. In this study, we observed that only the S263D mutant protein, not WT zM6Ab, could induce neurite outgrowth in zebrafish embryos (Fig. 5A, panels b and c). This suggests that the WT zM6Ab needs to be activated by unknown protein kinases to promote neurite outgrowth in zebrafish embryos. To explore which protein kinase has the potential to activate the WT zM6Ab, we generated a constitutively active CaMKIIβ1 (T287D) from zebrafish through site-directed mutagenesis in accordance with a previous report that mammalian CaMKIIβ can regulate neurite extension in rat hippocampal neurons [35]. Interestingly, enhanced neurite outgrowth by zM6Ab-GFP was observed in zebrafish embryos in the presence of CaMKIIβ1 (T287D)-DsRed (Fig. 5B). But the level was less than that of the S263D mutant protein. This suggests that other protein kinases in addition to zCaMKIIβ may regulate phosphorylation of zM6Ab in zebrafish embryos to induce neurite outgrowth.

In mammals, dendritic spines in the hippocampus are small protrusions from the main dendritic stalk with important roles in learning and memory [36]. Calcium signaling can modulate the activity of many proteins implicated in neurite, filopodium, and spine formation [37], [38]. In the present study, zebrafish M6Ab showed its ability to induce filopodium formation in PC12 cells (Fig. 2) and to promote neurite outgrowth in zebrafish embryos (Fig. 5). As for spine formation, the S263D mutant protein of zM6Ab will be an ideal target to test once a hippocampus-specific or midbrain-specific promoter is available which can drive the expression of this gene.

## Methods

### Zebrafish care

Zebrafish embryos were raised at 28.5°C, and different developmental stages were determined based on criteria described in the *Zebrafish Book* [39]. All animal handling procedures were approved by the Animal Use and Care Committee of Academia Sinica (protocol #10-12-114).

### Reagents and antibodies

U0126 (1,4-diamino-2,3-dicyano-1,4-bis[2-aminophenylthio] butadiene) and KN-62 were obtained from Sigma (St Louis, MO, USA). N-glycosidase F was purchased from Roche (Indianapolis, IN, USA). mNGF 2.5S (mouse nerve growth factor 2.5S) was acquired from Promega (Madison, WI, USA). A mouse monoclonal HA-probe (F-7) was obtained from Santa Cruz Biotechnology (Santa Cruz, CA, USA). Cy2-conjugated secondary antibodies were purchased from Jackson Immunoresearch Laboratories (West Grove, PA, USA).

### Cell culture and plasmid transfection

Monkey kidney fibroblast COS-1 cells (ATCC CRL-1650; Manassas, VA, USA) were cultured in high-glucose Dulbecco's modified Eagle's medium (DMEM), supplemented with 10% fetal bovine serum (FBS; Hyclone, Logan, UT, USA) in a humidified atmosphere of 5% CO_2_ at 37°C. Rat PC 12 cells were purchased from ATCC (CRL-1721; VA, USA) and cultured in low-glucose DMEM, supplemented with 10% FBS in a humidified atmosphere of 5% CO_2_ at 37°C.

PC12 and COS-1 cell transfection was conducted using the PolyJet In Vitro DNA Transfecton Reagent (SignaGen Laboratories, Ijamsville, MD, USA) following the manufacturer's instructions. COS-1 or PC12 cells were transfected with pcDNA3.1-GFP or pcDNA3.1-M6Ab-GFP. Transfected cells were then harvested at 24 and 48 h, fixed with 4% paraformaldehyde, and permeabilized in PBS with 0.1% Triton X-100. In order to block CaMKII kinase or mitogen-activated protein kinase (MAPK) kinase 1/2 (MEK1/2) activities, transfected cells were then exposed to U0126 (10 mM) or KN-62 (10 mM) for 72 h, respectively. The kinase inhibitors were first dissolved in dimethyl sulfoxide to a concentration of 1 mM and then diluted to 1/100 with culture media before use.

### Immunostaining

Immunostaining was performed using an anti-HA monoclonal antibody (1∶500 dilution) at 4°C overnight, followed by incubation with a Cy2-conjugated goat-anti-mouse antibody for 30 min at room temperature. Photo images were captured with a Zeiss LSM510 laser scanning confocal microscope (Carl Zeiss, Jena, Germany).

### Peptide: N-glycosidase F (PNGase F) treatment

COS-1 cells in 100-mm plates were grown to 80% confluence and transfected with plasmids encoding the WT M6Ab-HA fusion protein or M6Ab T166A mutant protein (8 µg of DNA) using the PolyJet In Vitro DNA Transfection Reagent (SignaGen Laboratories) following the manufacturer's instructions. Forty-eight hours after transfection, cells were harvested and lysed in 0.4 ml lysis buffer (150 mM NaCl, 20 mM HEPES (pH 7.2), 10 mM NaF, 1 mM EDTA, 0.5% NP-40, 1 mM Na_3_VO_4_, 1 mM PMSF, and 1 mM DTT), and incubated for 30 min at 4°C. The lysate was centrifuged at 13,000 rpm for 15 min. The concentrated supernatant was digested with 1 U of PNGase F (Roche, Indianapolis, IN, USA) at 37°C for 18 h. Untreated and N-glycosidase-treated culture supernatants were separated by sodium dodecylsulfate polyacrylamide gel electrophoresis (SDS-PAGE) and were then electrophoretically transferred to polyvinylidene difluoride (PVDF) membranes according to the Western blot method.

### Isolation of the full-length *M6Ab* and *CaMKIIβ1* from zebrafish

Complementary (c)DNAs encoding the complete open-reading frame (ORF) of zebrafish *M6Ab* and CaMKIIβ1 were obtained by PCR amplification according to the NCBI GenBank database with the respective accession nos. of AB089242 and XM_685461. The primers used were as follows: M6Ab forward primer, 5′-AAA AGC TTA TGG AAG AGA ACA TGG AAG AG -3′ and reverse primer, 5′-GGG GTA CCT GTG TAT GCG TTC AGG CGC TC-3′ and CaMKIIβ1 forward primer, 5′-CGG GAA GAC ATG GCC ACG ACT ACA TGT-3′ and CaMKIIβ1 reverse primer, 5′-TAG ATG TTG CTA CAA TGA GCT CAA CCT-3′.

### Site-directed mutagenesis of zebrafish M6Ab

Site-directed mutagenesis was performed to generate plasmids encoding M6Ab mutants such as T166A, S263A,S263D, S274A/S277A, S274D/S277D, A3(S263A/S274A/S277A), D3(S263D/S274D/S277D), and the constitutive form of CaMKII · β1(T287D) using the pGEM-T-M6Ab and pGEM-T-CaMKIIb plasmids as templates in the Quick Change Site-Directed Mutagenesis kit (Stratagene, La Jolla, CA, USA) according to the manufacturer's instructions. The corresponding oligonucleotides used were as follows (with the altered bases underlined): T166A-F: 5′-AAC ACT TGT CAG AAC ATG ACT CTG CTG GAG-3′, T166A-R: 5′-CTC CAG CAG AGT CAT GTT CTG ACA CGT GTT-3′, S263A-F: 5′-GAG GAC ATC AAG GCC AAG GAG GAG C-3′, S263A-R: 5′-GCT CCT CCT TGG CCT TGA TGT CCT C-3′, S263D-F: 5′-AGG ACA TCA AGG ACA AGG AGG AGC-3′, S274A/S277A-F: 5′-ATC CAC GCT ACT CGC GCT AAA G-3′, S274A/S277A-R: 5′-CTT TAG CGC GAG TAG CGT GGA T-3′, S274D/S277D-F: 5′-ATC CAC GAT ACT CGC GAT AAA G-3′, S274D/S277D-R: 5′-CTT TAT CGC GAG TCT CGT GGA T-3′, CaMKII · β1(T287D)-F: 5′-AGA CAG GAG GAT GTG GAA TGC CTG-3′, and CaMKII · β1(T287D)-R: 5′-CAG GCA TTC CAC ATC CTC CTG TCT-3′. The sequences of the resultant plasmids were verified using DNA sequencing.

### Construction of expression plasmids

To express the GFP fusion proteins or HA-tagged proteins in PC12 and COS-1 cells, cDNA encoding each of the M6Ab and M6Ab mutants was re-amplified by a PCR using primers with HindIII and KpnI restriction sites followed by subcloning of the PCR products into pcDNA-GFP, pcDNA-DsRed, or pcDNA-HA to generate pcDNA-M6Ab-GFP, pcDNA-M6Ab-D3-GFP, pcDNA-CaMKII · β1 (T287D)-DsRed, pcDNA-M6Ab-HA, pcDNA-M6Ab(T166A)-HA, pcDNA-M6Ab(S263A)-HA, pcDNA-M6Ab(S263D)-HA, pcDNA-M6Ab(S274A/S277A)-HA, pcDNA-M6Ab(S274D/S277D)-HA, pcDNA-M6Ab(A3)-HA, and pcDNA-M6Ab(D3)-HA.

To express GFP fusion proteins and DsRed fusion proteins in neurons, each DNA fragment encoding the GFP-fusion or DsRed-fusion protein from pcDNA-M6Ab-GFP, pcDNA-M6Ab-A3-GFP, pcDNA-M6Ab-D3-GFP, and pcDNA-CaMKII · β1(T287D)-DsRed respectively, was released by the BamHI and XhoI sites, and inserted into the corresponding sites of the pHuC-GFP plasmid to replace the GFP coding region and respectively generate pHuC-M6Ab-GFP, pHuC-M6Ab-D3-GFP, and pHuC-CaMKII · · 1(T287D)-DsRed. The control plasmid, pHuC-GFP or pHuC-DsRed [18], [19], was previously described, and GFP or DsRed genes were driven by a zebrafish neuron-specific HuC promoter [17].

### Protein in-gel digestion

The protein bands on 1D gel were manually excised from the gel and cut into pieces. The gel pieces were dehydrated with acetonitrile for 10 min, vacuum dried, rehydrated with 50 mM DTE in 25 mM ammonium bicarbonate, pH 8.5, at 37°C for 1 h, and subsequently alkylated with 100 mM iodoacetamide in 25 mM ammonium bicarbonate, pH 8.5, at room temperature for 1 h. The pieces were then washed twice with 50% acetonitrile in 25 mM ammonium bicarbonate, pH 8.5 for 15 min each time, dehydrated with acetonitrile for 10 min, dried, and rehydrated with a total of 10 ng of sequencing grade, Asp-N (Promega, Madison, WI, USA) in 25 mM ammonium bicarbonate, pH 8.5, at 37°C for 16 hr. Following digestion, digested peptides were extracted twice with 50% acetonitrile containing 5% formic acid for 15 min each time with moderate sonication. The extracted solutions were pooled and evaporated to dryness under vacuum.

### Enrichment of phosphopeptides from the digested sample

The phosphorylated peptides were concentrated using a Titansphere Phos-Tio kit (GL Sciences, Tokyo, Japan). Prior to loading samples, the titania tips were equilibrated with 0.1% TFA, 80% acetonitrile (solution A) and 300 mg/mL lactic acid in 0.1% TFA, 80% acetonitrile (solution B). The digested sample was diluted with 100 mL of solution B and loaded onto the titania tip. After successive washing with 20 mL each of solution B and solution A, 50 mL each of 5% ammonium hydroxide and 5% Pyrrolidine were used for elution. The eluted fraction was acidified with 20 mL of 1% TFA, desalted using C18 ZipTip and concentrated in a vacuum evaporator, followed by the addition of 0.1% FA for the subsequent nanoLC-MS/MS analysis.

### Nanoflow HPLC-MS/MS

The peptide mixtures were analyzed by online nanoflow liquid chromatography tandem mass spectrometry (LC-MS/MS) on a nanoAcquity system (Waters, Milford, MA, USA) connected to an LTQ Orbitrap Velos hybrid mass spectrometer (Thermo Fisher Scientific, Bremen, Germany) equipped with a PicoView nanospray interface (New Objective, Woburn, MA, USA). Peptide mixtures were loaded onto a 75 mm ID, 25 cm length C18 BEH column (Waters) packed with 1.7 mm particles with a pore with of 130 Å and were separated using a segmented gradient in 30 min from 5% to 40% solvent B (acetonitrile with 0.1% FA) at a flow rate of 300 nl/min and a column temperature of 35°C. Solvent A was 0.1% FA in water. The effluent from the HPLC column was directly electrosprayed into the mass spectrometer. The LTQ-Orbitrap Velos mass spectrometer was operated in positive ion mode and a data-dependent “Top 20” method was employed. In each cycle, a full-scan spectrum was acquired in the Orbitrap at a target value of 5E5 ions with resolution R = 60,000 at m/z 400 followed by ion-trap CID on the 20 most intense ions with a target value of 5E3 ions. The ‘lock mass’ function was enabled for the MS mode, where the background ion at m/z 391.284286 was used as the lock mass ion. General MS conditions were as follows: spray voltage, 1.9 kV; no sheath or auxiliary gas flow; S-lens, 50%. FT preview mode was enabled, charge-state screening enabled, and rejection of singly charged ions enabled. Ion selection thresholds were 500 counts for MS2, 35% normalized collision energy, activation q = 0.25, and activation time of 10 ms were applied for CID. Dynamic exclusion was employed and 10 ppm window of the selected m/z was excluded for 90 s. near ion trap. To improve the fragmentation spectra of the phosphopeptides, “multistage activation” at 97.98, 48.99, 32.66, and 24.49 Thompson (Th) relative to the precursor ion was enabled in all MS/MS events. The MS and MS/MS raw data were processed by Raw2MSM and searched against NCBI database (5/26/2011) with the Mascot Daemon 2.3 server. Search criteria used were AspN digestion, variable modifications set as carbamidomethyl (C), oxidation (M) and phosphorylation (STY) allowing up to 2 missed cleavage, mass accuracy of 10 ppm for the parent ion and 0.60 Da for the fragment ions. Phosphorylation sites and peptide sequence assignments contained in MASCOT search results were validated by manual confirmation from raw MS/MS data.

### Microinjection of the expression plasmid into zebrafish embryos

The expression plasmid was linearized by digestion with suitable restriction enzymes and purified with a PCR Gel extraction kit (Qiagen, Hilden, Germany). DNA was adjusted to a final concentration of 100 µg/ml in 1× Danieau solution (5 mM Hepes (pH 7.6), 58 mM NaCl, 0.7 mM KCl, 0.4 mM MgSO_4_, and 0.6 mM Ca(NO_3_)_2_) containing 0.5% phenol red and injected into zebrafish embryos at the one-cell stage using a Narishige IM 300 microinjector system (Narishigi Scientific Instrument Lab., Tokyo, Japan).

Embryos at 48 h post-fertilization (hpf) were observed under an Olympus IX70- FLA inverted fluorescence microscope. Images were taken using a Zeiss LSM510 laser scanning confocal microscope (Carl Zeiss).

### Statistical analysis

Quantitative data from three to three independent experiments are expressed as means (±SD). Unpaired Student's t-tests were used to analyze between group differences. P<0.05 was considered statistically significant.
